# Collective rotational motion of freely expanding T84 epithelial cell colonies

**DOI:** 10.1098/rsif.2022.0719

**Published:** 2023-02-22

**Authors:** Flora Ascione, Sergio Caserta, Speranza Esposito, Valeria Rachela Villella, Luigi Maiuri, Mehrana R. Nejad, Amin Doostmohammadi, Julia M. Yeomans, Stefano Guido

**Affiliations:** ^1^ Dipartimento di Ingegneria Chimica, dei Materiali e della Produzione Industriale (DICMAPI), Università di Napoli Federico II, P.le Tecchio 80, 80125 Napoli, Italy; ^2^ CEINGE Biotecnologie Avanzate, Via Sergio Pansini 5, 80131 Naples, Italy; ^3^ European Institute for Research in Cystic Fibrosis, San Raffaele Scientific Institute, Milan, Italy; ^4^ The Rudolf Peierls Centre for Theoretical Physics, Department of Physics, University of Oxford, Parks Road, Oxford OX1 3PU, UK; ^5^ The Niels Bohr Institute, University of Copenhagen, Copenhagen, Denmark

**Keywords:** epithelial cells, active matter, collective rotation, living matter, active nematics

## Abstract

Coordinated rotational motion is an intriguing, yet still elusive mode of collective cell migration, which is relevant in pathological and morphogenetic processes. Most of the studies on this topic have been carried out on epithelial cells plated on micropatterned substrates, where cell motion is confined in regions of well-defined shapes coated with extracellular matrix adhesive proteins. The driver of collective rotation in such conditions has not been clearly elucidated, although it has been speculated that spatial confinement can play an essential role in triggering cell rotation. Here, we study the growth of epithelial cell colonies freely expanding (i.e. with no physical constraints) on the surface of cell culture plates and focus on collective cell rotation in such conditions, a case which has received scarce attention in the literature. One of the main findings of our work is that coordinated cell rotation spontaneously occurs in cell clusters in the free growth regime, thus implying that cell confinement is not necessary to elicit collective rotation as previously suggested. The extent of collective rotation was size and shape dependent: a highly coordinated disc-like rotation was found in small cell clusters with a round shape, while collective rotation was suppressed in large irregular cell clusters generated by merging of different clusters in the course of their growth. The angular motion was persistent in the same direction, although clockwise and anticlockwise rotations were equally likely to occur among different cell clusters. Radial cell velocity was quite low as compared to the angular velocity, in agreement with the free expansion regime where cluster growth is essentially governed by cell proliferation. A clear difference in morphology was observed between cells at the periphery and the ones in the core of the clusters, the former being more elongated and spread out as compared to the latter. Overall, our results, to our knowledge, provide the first quantitative and systematic evidence that coordinated cell rotation does not require a spatial confinement and occurs spontaneously in freely expanding epithelial cell colonies, possibly as a mechanism for the system.

## 1. Introduction

The cooperative movement of cell groups, sheets or strands, which is referred to as collective cell migration [1], plays a key role in several physiological as well as pathological processes including morphogenesis, tissue repair, immune response and cancer progression [2–5]. In tumour invasion, for example, collective cell movement allows malignant tumour cells to escape the primary tumour and invade surrounding tissues [6,7]. Similarly to single cell migration, collective cell movement is mainly driven by actin polymerization and myosin-driven contractility coupled to cell polarity [8], but occurs under additional constraints, determined by cell–cell junctions and other close cell interactions [8,9]. The latter includes several processes that have been proposed to affect collective migration, such as direct cell–cell chemical signalling, physical interactions underlying the mechanical integrity of clusters, the coordinated polarization of leader cells on cluster edges possibly guiding the behaviour of follower cells, and the secondary remodelling of the extracellular matrix along the migration track [8,10]. Traction force mapping shows long-range force transmission within sheets or clusters in a cooperative way: each cell, at the leading edge as well as inside, takes part in a global ‘tug-of-war’ through cell–cell junctions that maintains the collective structure in a global state of tensile stress [11–13]. Physical signals from the substrata, such as local rigidity [14], have also an effect on cell migration.

The complex interplay between different processes over a wide range of spatio-temporal scales makes the interpretation of cooperative cell behaviour a challenging task. Some important insights have been provided by suggesting several analogies with other phenomena, such as the flow of multiphase fluids (e.g. the spreading and coalescence of droplets [14]), the mechanical behaviour of liquid crystals [15,16] and the rheology of the glassy state [17]. These analogies are based on the fact that cell–cell junctions are fluid in the sense that they can be mutually displaced, thus allowing cell motion even in dense aggregates. Hence, one can describe cell motility in analogy with temperature-driven molecular motion and introduce a diffusion coefficient to model cell migration against a cell density gradient. However, two important differences arise with respect to the case of ordinary fluids: cells are able to propel themselves in a given direction and can proliferate, which are two typical attributes of living active matter [18]. Therefore, collective cell migration can be considered as the result of three main effects: cell proliferation, diffusive migration and directional motion. The latter can be elicited by some substrate anisotropy, such as chemotaxis and contact guidance.

A mode of collective cell migration that has attracted much interest in the literature is the coordinated rotation of cells, also referred to as coherent angular motion (CAM) [19]. Such rotational motions have been observed in human breast epithelial cells cultured in laminin-rich gels *in vitro* and interpreted as an essential mechanism for the formation of acini, polarized spherical structures with basolateral and apical membrane regions around a central lumen found in mammary glands [19]. Lumen formation *in vitro* by Madin–Darby canine kidney (MDCK) epithelial cells aggregates has also been associated with circular cell motility [20], with a rate decreasing with increasing cell number, suggesting a transition to epithelial polarization during aggregate development [21]. *In vivo*, collectively rotating cell structures have been observed during morphogenesis, such as in the development of the primitive streak in gastrulating chick embryos [22].

Most recent studies on collective cell migration, including CAM, have been carried out by constraining cell motion inside confined regions obtained by micropatterning islands coated with extracellular matrix adhesive proteins (e.g. [18,23–27]). As opposed to the classical wound healing assay, where the time taken by the cells to fill a gap created by scratching a confluent cell monolayer is measured as an index of collective cell migration [28], the technique of micropatterning allows a better control of domain geometry both in terms of size and shape (e.g. circular and rectangular regions of different size can be created). In addition, cell motion inside micropatterned surfaces can be investigated by several techniques, such as time-lapse microscopy, epifluorescence and confocal imaging, particle image velocimetry and image analysis, enabling spatio-temporal maps of cell position, orientation and shape with subcellular resolution to be obtained. This experimental characterization can be compared with theoretical modelling of collective cell migration. The latter has been based on several approaches, including continuum modelling by the Fisher–Kolmogorov equation [26,29], phenomenological migration motives [30,31] (e.g. plithotaxis and kenotaxis), and the already mentioned analogies with glassy dynamics [17], droplet-like spreading on rigid surfaces [14,32] and active nematic liquid crystals [33–35].

Under confined conditions in circular patterns, MDCK cells are found to exhibit solid-body behaviour of synchronized collective rotation when they reach confluency [36,37]. It has been speculated that such behaviour is initiated by cells at the border of the circular regions, which are guided by the edge and tend to transmit their directional motion to inner cells through cell–cell contacts [36]. Indeed, by downregulating intercellular adhesion the synchronized collective cell rotation is reduced. Cell–cell contacts provide directional guidance to neighbour cells, so that the ring orientation at the periphery of the circular patterns is propagated to inner cells. This mechanical coupling is altered by the presence of a cell in the centre of the circular pattern owing to the lack of a stable axis of internal polarization, which can explain the discontinuity in the persistence time of CAM between 4 and 5 cells (when a central cell is observed in the circular pattern) [38]. Another factor playing a key role in CAM is cell division, which has been shown to induce extensile forces and turbulent-like velocity fields in confluent cell monolayers [33,39]. Cell division is also associated with a switching of the direction of collective cell rotation [39]. Blocking cell proliferation with mitomycin-C impairs CAM and the switches of the direction of rotation. Division of a cell located near the periphery is more effective in inducing the onset of CAM as compared to the division of inner cells [39].

Although much progress has been made in the understanding of collective cell rotation, there are still several aspects to be fully elucidated. In particular, while some consensus has been reached on the role of confinement in inducing CAM, the same does not apply to the driver of such cell collective behaviour, for which an intriguing explanation could be spontaneous symmetry breaking. In principle, some further insight on collective cell rotation could be obtained by looking at unconstrained expansion of cell colonies, which has been less studied in the literature as compared to the case of confined conditions. In [40], it is reported that an acto-myosin cable in the outer boundary of the colonies could lead to rotational motion of cells at the periphery of the colony. In another work addressing the topic of freely expanding epithelial MDCK cells [41], it has been shown that in the first 5–6 days the area of a colony grows according to a simple exponential law while cell density remains constant. Furthermore, cells move outwards on the average, with non-uniform velocity at the periphery and finger-like protrusions. At a critical value of area, the outwards expansion of the colony cannot keep pace with cell proliferation and cell density starts increasing. At this point, a transition to an epithelial morphology is observed, where cell height is increased, local ordering appears in the colony and cell proliferation and motion are strongly inhibited [41]. Overall, these results provide a quantitative characterization of collective cell migration and growth in freely expanding cell colonies, but do not address coordinated cell rotation.

Indeed, data on coordinated cell rotation in this free growth regime are still lacking in the literature and the main objective of our work is to study such phenomenology. Here, we focus on collective rotation in freely expanding epithelial colonies of the T84 intestinal cell line, where, at variance with micropatterned substrata, cell clusters can grow with no geometrical constraints. The T84 cells are seeded in multi-well culture plates and the evolution of the growing cell clusters is followed up to confluency by time-lapse video microscopy and image analysis. The latter allows us to track the position of individual cells as a function of time and to investigate their angular motion inside the clusters. The results of this work are relevant in several fields, from tissue morphogenesis to the repair of injuries of the epithelial layer lining the intestinal lumen.

## 2. Material and methods

### 2.1. Cell cultures

Human colon adenocarcinoma T84 cells were cultured in Dulbecco’s modified Eagle medium F12 supplemented with 10% (v/v) fetal bovine serum and antibiotics ($50\;{\mathrm{units}\;\mathrm{ml}}^{-1}$ penicillin and 50 μg ml^−1^ streptomycin) and maintained in a humidified incubator at 37°C under an atmosphere of 5% CO_2_ in air.

### 2.2. Time-lapse microscopy

T84 cells were plated on 24 multi-well culture dishes at varying cell density. The multi-well plate was placed on the stage of an inverted microscope (Zeiss Axiovert 200) equipped with motorized sample positioning and focusing (Marzhauser). The microscope was enclosed in a plastic cage to control environmental conditions (temperature: 37°C, CO_2_ concentration: 5%, humidity level: close to saturation) [28,42]. Live cell phase contrast imaging was performed by a high-resolution monochromatic CCD video camera (Hamamatsu Orca AG). Microscope operations were controlled by a time-lapse software allowing us to select multiple fields of view and the time interval between consecutive image acquisitions during the experiment [43,44]. To follow T84 collective behaviour, images were iteratively acquired using a 5 × objective at several locations within the culture dish, with an image acquisition frequency of 2 h; the overall experimental length was 14 days. The cells were rinsed with fresh culture medium every 2 days, without removing the culture dish from the microscope stage. To capture the dynamic behaviour of individual cells within the aggregates, images were iteratively acquired using a 20 × objective. Acquisition frequency was 10 min, the experiment length was 70 h.

### 2.3. Image analysis

The number of cells (*N*_cells_) within each cell cluster was determined by using the Image Pro Plus analysis software, which allows us to manually count the cells in a selected region. The same software was used to measure the area of the cell clusters (*A*_cluster_) by manually tracing their contour in the image overlay. The average area of individual cells inside a cluster (*A*_cell_) was determined as the ratio between the area of the cluster (*A*_cluster_) and the number (*N*_cells_) of enclosed cells.

By using semiautomatic routines (ImageJ), the *X* and *Y* coordinates of the centroid of individual cells were manually measured at each time step, and the corresponding cell trajectories were reconstructed. In order to quantify cell movement in a cluster, the radial (*V*_*ρ*_) and angular (*ω*) cell velocity were calculated from the net cell displacement with respect to the cluster centroid, along the radial and the angular direction, respectively, over a time interval of 2 h. The data were then averaged over the entire cell population. The angles were measured counterclockwise and cell tracking was done for 12 h.

### 2.4. Equations of motion for the continuum model

Active nematic descriptions have been very successful in describing the dynamics of cell monolayers [45–47]. The fundamental continuum equations that describe wet active nematics (active nematohydrodynamic equations) are coupled equations for the evolution of the cell concentration *ϕ*, nematic tensor, **Q** = *S*(**nn** − **I**/2) in two dimensions and the associated incompressible fluid velocity, **u**. They read:

2.1 $$\partial_{t}Q+u\cdot \nabla Q-W=\gamma \;H,$$

2.2 $$\rho (\partial_{t}+u\cdot \nabla )u=\nabla \cdot \Pi ,\;\nabla \cdot u=0,$$

2.3 $$\partial_{t}\phi +u\cdot \nabla \phi =\Gamma_{\phi }\nabla^{2}\mu ,$$

2.4 $$\mu =\frac{\partial f}{\partial \phi }-\nabla \cdot \left(\frac{\partial f}{\partial \nabla \phi }\right),$$

2.5 $$f=-\frac{C}{2}{\left(1+Q_{ij}Q_{ij}/2\right)}^{2}+\frac{B}{2}\phi^{2}(1-\phi^{2})$$

2.6 $$+\frac{K_{\phi }}{2}\nabla_{m}\phi \nabla_{m}\phi +\frac{K}{2}\nabla_{m}Q_{ij}\nabla_{m}Q_{ij}.$$

In the definition of the nematic tensor the director field **n** represents the orientation of the nematic alignment and the magnitude of the nematic order is denoted by *S*. In the evolution of the **Q** tensor, equation (2.1), *γ* is the rotational diffusivity and the molecular field, H=−∂f/∂Q+∇⋅(∂f/∂∇Q), drives the nematic tensor towards the minimum of a free energy density *f*. The generalized advection term:

2.7 $$W=(\lambda E+\Omega )\cdot \left(Q+\frac{I}{2}\right)+\left(Q+\frac{I}{2}\right)\cdot (\lambda E-\Omega )$$

2.8 −λ(Q+I)Tr(Q⋅E),

models the response of the nematic field to the strain rate **E** and vorticity Ω, where *λ* is the flow-aligning parameter. In the Navier–Stokes equation (2.2), *ρ* is the density of the suspension, and the stress tensor, Π, includes viscous, elastic and active contributions. The viscous stress, $\Pi^{v}=2\eta E$, where *η* is the viscosity, and the elastic stress:

2.9 $$\begin{array}{rl} \Pi_{ij}^{\;p} & =-P\delta_{ij}+\lambda (Q_{ij}+\delta_{ij})Q_{kl}H_{kl}-\lambda H_{ik}\left(Q_{kj}+\frac{\delta_{kj}}{2}\right)\\ & -\lambda (Q_{ik}+\frac{\delta_{ik}}{2})H_{kj}+Q_{ik}H_{kj}-H_{ik}Q_{kj}\\ & -K(\partial_{i}Q_{kl})(\partial_{j}Q_{kl}), \end{array}$$

where *P* is the pressure, are familiar terms that appear in the dynamical equations of passive liquid crystals. Coarse-graining the dipolar force fields of the active nematogens leads to an active contribution to the stress that characterizes wet active nematics, $\Pi^{a}=\zeta Q$. In extensile systems (*ζ* < 0), it acts to extend a nematic region along its director whereas in contractile materials (*ζ* > 0) it contracts a nematic region along the director [48]. Equation (2.3) describes the evolution of the concentration of the active material, where $\Gamma_{\phi }$ shows how fast *ϕ* responds to gradients in the chemical potential *μ*.

The first term in the free energy density (equation (2.6)) leads to the isotropic phase so that in our system, any nematic order is caused by activity [49,50]. The second and third terms lead to the formation of cell clusters (identified by *ϕ* = 1) in a cell-free background (*ϕ* = 0), where *C*, *B* and *K*_*ϕ*_ are material parameters. The last term in equation (2.6) represents the energy cost owing to distortions in the nematic field, assuming a single Frank elastic constant *K*.

The continuum equations of motion are solved using a hybrid lattice Boltzmann and finite difference method [51–53].

### 2.5. Simulation parameters

For the simulations, we used a box of size 300 × 300 in lattice Boltzmann units with periodic boundary conditions, and start the simulations with circular drops of various sizes with a random director (the initial orientation is randomly chosen and is in the interval [0, 2*π*]). We used the parameter values *ρ* = 40, $\Gamma_{\phi }=0.2$, *γ* = 0.3, |*ζ*| = 0.01, |*χ*| = 0.7, *K* = 0.02, *K*_*ϕ*_ = 0.1, *B* = 0.01, *C* = 0.001. Since it is not possible to find the value of the simulation parameters using the experimental data, we have used a set of the parameters that in an unconfined large system gives us patterns that have been observed in different experiments on living systems. These include the formation and annihilation of defects, their self-propulsion, and the velocity field that they produce [45,49]. To compare the rotational velocity as a function of area with the experimental data, we matched the initial area and angular velocity in simulations with the initial area and angular velocity in the experiment and rescaled the axis accordingly.

## 3. Results and discussion

Previously some of us have investigated the dynamics of monolayer formation in human colon adenocarcinoma T84 cells *in vitro* by using time-lapse microscopy [42]. As summarized in the Introduction, the expansion of an epithelial colony is generally driven by a complex interplay of proliferation [54], cell growth [41], motility [55] and cluster fusion mechanisms [56]. At early times upon seeding T84 cells in culture plates, two-dimensional cell clusters or islands of different size, depending on the initial cell density, were observed. Initially, cell clusters were separate from each other and their size increased in time without apparent mutual interactions. As time went on, however, cell clusters came in contact with each other during their growth and merged together in larger clusters, up to the eventual formation of a cell monolayer spanning the entire available surface of the culture plate. It was also observed that cells at the cluster boundary extended long filopodia reaching out to nearby clusters before their merging. A quantitative characterization of the expansion and growth of T84 cell clusters up to the formation of a continuous monolayer was carried out by measuring the number of cells within the clusters and the cluster size as a function of time. Furthermore, the motility of single cells within the clusters was analysed as described in the previous section.

### 3.1. Cell proliferation analysis

**Figure 1.**
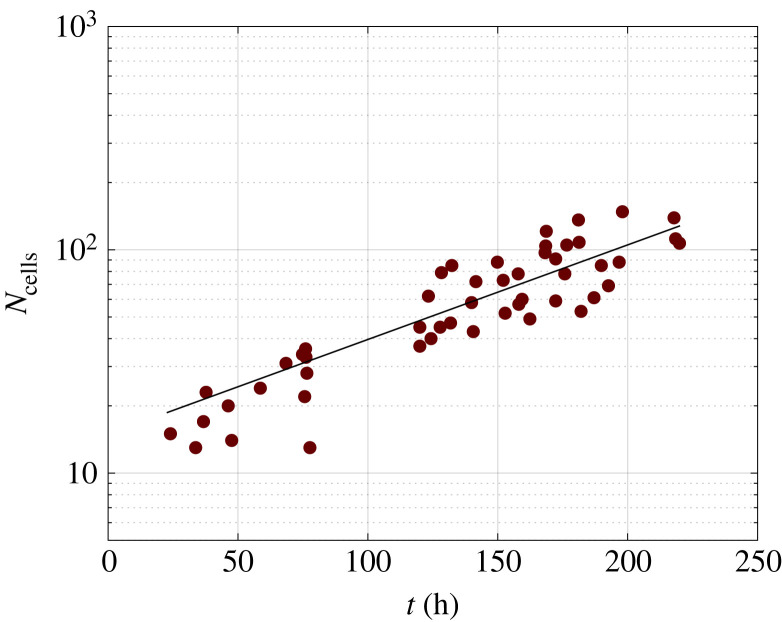
Dynamic evolution of the number of cells within the clusters. The number of cells (*N*_cells_) is plotted as a function of time. An exponential fit is shown as a continuous line $\log\;(N_{\mathrm{cells}}(t))=\log(N_{0})+(t/t_{d})\;\log\;2$.

In figure 1, the number of T84 cells inside a cluster, *N*_cells_, is plotted as a function of time. It can be noted that data from different cell clusters are pooled together in the plot of figure 1 and, apart from some scatter, they seem to follow the same trend. The latter is well represented by a simple exponential law:

3.1 $$N_{\mathrm{cells}}(t)=N_{0}2^{t/t_{d}},$$

where *t* is the time from cell plating, *N*_cells_(*t*) is the number of cells at time *t*, *N*_0_ is the number of cells at time 0 and *t*_*d*_ is the cell duplication time. Equation (3.1) was fitted to the experimental data in figure 1, with *t*_*d*_ as the only adjustable parameter. This approach neglects the contribution of cell death to the cell population balance, assuming that it is not relevant in the initial phase of cell growth. The exponential fitting curve is shown as a continuous line in the graph in figure 1. Based on the fit, the duplication time of the cells within the clusters was estimated to be 71 h (with *R*_2_ = 0.71 and a standard error of estimate of 19 h). Figure 1 shows that the number of cells as a function of time increases linearly with time. This does not follow the classical logistic cell growth, in which cell division rate decreases in time. In fact, a logistic cell growth pattern is characterized by a sigmoidal shape with a decreased rate when cell density becomes so large that further proliferation is inhibited. The time frame of the experiments is indeed up to the onset of confluency and the experiments are not carried out afterwards (see next section), since our focus is to study coordinated cell rotation in isolated cell clusters, i.e. well before confluency.

### 3.2. Growth of cell clusters

**Figure 2.**
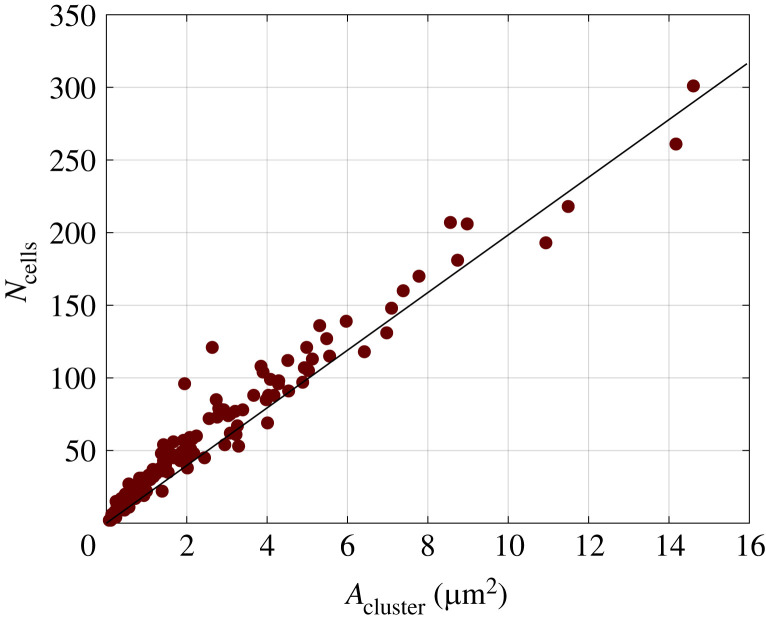
The number of cells (*N*_cells_) within the clusters is plotted as a function of the cluster area (*A*_cluster_). The continuous line is a linear fit to the data *N*_cells_ = 19.7 *A*_cluster_.

The morphology of cell clusters was studied by manual tracing of their boundaries followed by application of image analysis routines (see Material and methods). The resulting cell cluster area, *A*_cluster_, is correlated to *N*_cells_, the number of cells within a cluster, in figure 2, where results corresponding to 51 clusters are presented and the continuous line is a linear fit to the data. The plot in figure 2 shows a direct proportionality between *A*_cluster_ and *N*_cells_, thus implying that the average cell density in a cluster is constant in the time frame investigated. This finding is consistent with the so-called free expansion regime, where the increase of cluster area is governed by mitosis and each daughter cell takes an area equal to that of the mother cell [41].

As for *N*_cells_, the cluster area also grows exponentially (data not shown for the sake of brevity). The area exponential growth cannot continue indefinitely, since it would require an unbound exponential increase of cell velocity. At some point, cell velocity cannot keep up with area growth owing to cell proliferation, and cell density starts increasing, which marks the onset of the proliferation inhibition mentioned in the previous section. Following Puliafito *et al.* [41], the critical cluster area *A*_*c*_ at the transition between these two regimes can be estimated by the equation *v*_*ρ*_ = (*A*_*c*_/(4*πτ*^2^))^1/2^, where *v*_*ρ*_ is the radial cell velocity at the boundary and *τ* is the characteristic time of the exponential area growth. From this equation and the values *τ* = *t*_*d*_ = 71 h (see previous section) and *v*_*ρ*_ = 0.04 μm min^−1^ = 2.4 μm h^−1^ (see the cell motility section), one obtains *A*_*c*_ ∼ 4 × 10^5^ μm^2^ , which is above the largest value (1.5 × 10^5^ μm^2^) of cluster area in figure 2, thus confirming that the range explored in our experiments is within the free expansion regime.

**Figure 3.**
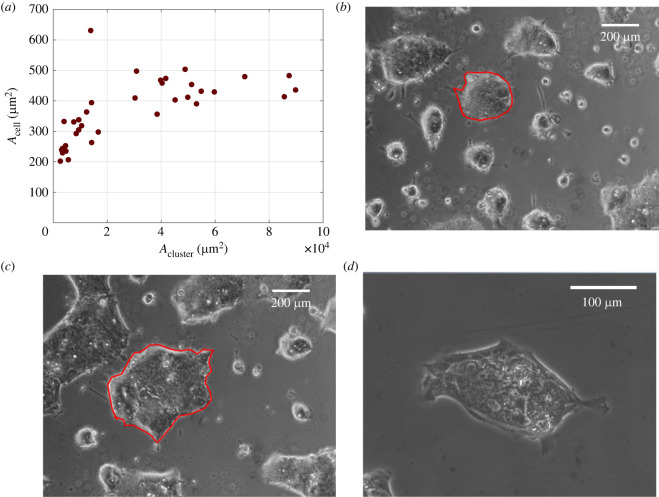
Average cell area is plotted as a function of cluster area (*a*). Two images of the same field of view at *t* = 0 (*b*) and *t* = 24 h (*c*) are shown below the plot. The contour of a cell cluster is highlighted as a red overlay in both images (scale bar = 200 μm). A larger view of a cell cluster showing the different morphology of cells at the edge and in the core (scale bar = 100 μm) is presented in (*d*).

A further analysis of cell density was performed by dividing cluster area by the corresponding number of cells and plotting the resulting average cell area *A*_cell_ as a function of cluster area *A*_cluster_, as shown in figure 3. It can be noted that *A*_cell_, which is the reciprocal of cell density, is constant with cluster area for cluster area higher than 2 × 10^4^ μm^2^. In the case of smaller clusters, where the data of cell number in figure 2 are slightly above the linear fit, *A*_cell_ is growing with *A*_cluster_. This trend can be explained by the spreading of cells at the boundary, as discussed in the following. The time evolution of clusters can be monitored by time-lapse video microscopy of the growing colony, as illustrated in the images at the bottom of figure 3. The image in figure 3*b*, corresponding to the beginning of the time-lapse experiment, is characterized by smaller clusters with bright boundaries in phase contrast, indicating thicker cells.

The clusters in figure 3*b*, corresponding to a time of 24 h after the beginning of the time-lapse experiment, are less bright at the boundary owing to the spreading of the cells therein. Several cell protrusions, such as filopodia and lamellipodia, between nearby clusters can be observed in both images of figure 3*b,c*, and provide a further argument in favour of the special role played by cells at the boundary. The image in figure 3*d* shows a larger view of a single cluster and it can be observed that cells at the boundary are more elongated and flattened as compared to cells in the middle. Such different morphology has been linked to the hypothesis that cells at the periphery can act as leaders dragging along the cells which are located inside the clusters, although this view is still a matter of debate [57]. Another possible reason for the difference of morphology is ‘active anchoring’—in an active extensile system the cells would tend to elongate parallel to the boundary [58].

### 3.3. Cell monolayer formation

**Figure 4.**
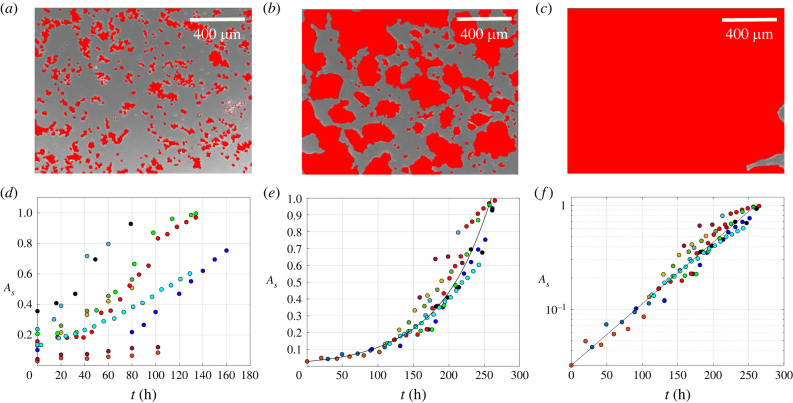
(*a*)–(*c*) Images of monolayer spreading at time *t* = 0 (*a*), *t* = 80 h (*b*) and *t* = 160 h (*c*), corresponding to *A*_*s*_ = 0.10, *A*_*s*_ = 0.50 and *A*_*s*_ = 0.99, respectively, are shown, with the area occupied by the cells highlighted in red. This is different from the scale shown on the image: scale bar is equal to 400 μm. Dynamics of monolayer formation for different initial cell densities. Cell confluence (*A*_*s*_) is plotted as a function of time for each cell sample under investigation. On the left (*d*) raw data from each sample are reported, on the right (*e*) the same data are shifted in time to generate a master curve, that can be used to estimate the characteristic time of the process. (*f*) The cell confluence plotted on a log scale as a function of time on a linear scale.

In addition to the growth of single-cell clusters, the evolution of the T84 colony is also affected by merging of cell clusters into larger aggregates, which eventually leads to a continuous cell monolayer spanning the entire plate surface. As illustrated by the images in figure 4*a*–*c*, the dynamics of monolayer formation was investigated by calculating, at each time step, a confluence parameter as the ratio (*A*_*s*_) between the area occupied by the cells in the image (red regions) and the size of the whole image. The images correspond to *t* = 0 (*a*), *t* = 80 h (*b*) and *t* = 160 h (*c*), for one of the cell samples. In figure 4*d*, *A*_*s*_ is plotted as a function of time for several cell cultures with different initial cell density, corresponding to different values of *A*_*s*_ at time 0. Each curve follows an exponential trend, up to almost 100% confluence (*A*_*s*_ = 1).

The curves in figure 4*d* share a common increasing trend as a function of time but appear as unrelated to each other because each cell culture starts from a different initial condition in terms of cell density. This difference can be taken into account by shifting the curves horizontally to find a possible superposition of the data onto a single master curve by matching the *A*_*s*_ values. As shown by the plot of figure 4*e*, the horizontal time shift leads indeed to the data collapse onto a master curve, thus showing that cells plated out with different initial densities (different *A*_*s*_) grow according to the same law, and the velocity of the process depends only on the actual value of *A*_*s*_. The master curve can be fitted by the following exponential function:

3.2 $$A_{s}(t)=A_{s0}2^{t/\tau },$$

where *t* is the time rescaled according to the horizontal shifting, *A*_*s*_(*t*) is the confluence at time *t*, *A*_*s*0_ is the initial confluence parameter at time *t* = 0 and *τ* is the doubling time of cell occupancy. Data from the master curve were fit according to equation (3.2) with *τ* as the only adjustable parameter (continuous line in figure 4). The so obtained value of *τ* was found to be 50.8 h, which is within the standard error of the characteristic time of the growth of single-cell clusters (71 ± 19 h, see figure 1). Once again, it can be noted that the simple exponential trend is different from the sigmoidal shape of a classic logistic growth owing to the limited time span of the experiment, which does not encompass the eventual levelling off of the data.

### 3.4. Cell motility

**Figure 5.**
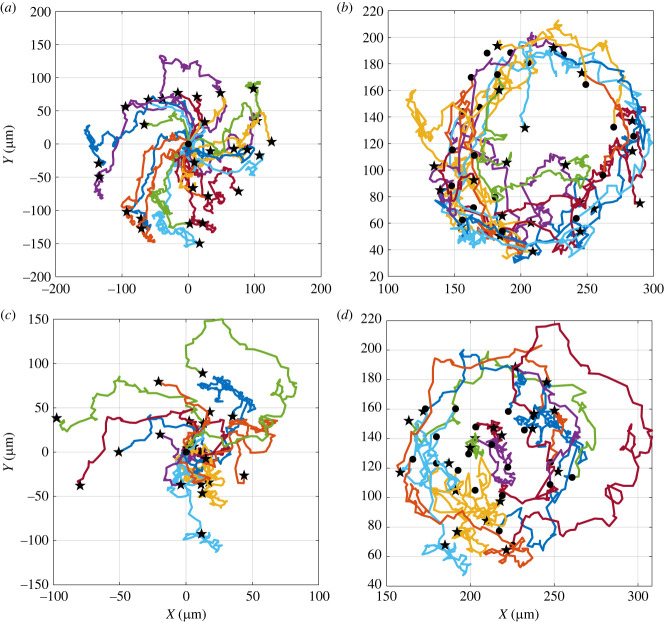
Trajectories of the cells moving in the outer (*a*,*b*) and inner (*c*,*d*) region of the cluster with an average radius of 84 μm and an aspect ratio around 0.83. Solid circles (stars) show the starting (end) points of the trajectory. A video of cluster rotation where trajectories of cells are superimposed for visual comparison is available in the electronic supplementary material, Movie 1.

We investigated the movement of individual cells inside a cluster as a function of their position. The clusters were divided into concentric regions to evaluate possible differences in the movement of the cells in the inner and outer region of the same colony. As an example, in figure 5 we tracked the positions of the cells moving in an almost circular cluster with an average radius of 84 μm and an aspect ratio (minor axis/major axis ratio) about 0.83. The cluster was divided into two regions, the inner one containing 23 cells with a position at the initial frame of the analysis within 46 μm from the cluster centre, and the external one containing 29 peripheral cells. In figure 5, the paths of the cells moving in the outer (*a*,*b*) and inner (*c*,*d*) regions are plotted in a frame of reference with the origin coincident with the initial cell position (figure 5*a*,*c*) or in the laboratory frame (figure 5*b*,*d*). The trajectories described by the cells in both regions show a circular shape, suggesting that the cells generate a coordinated collective rotation within the cluster.

**Figure 6.**
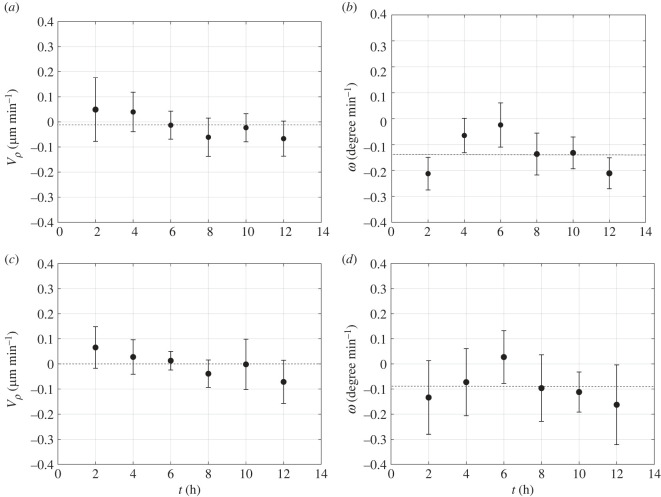
Evolution in time of the radial (*V*_*ρ*_), and angular (*ω*) velocity of the cells moving in the outer (*a*,*b*) and inner (*c*,*d*) region of a cluster with an average radius of 84 μm and an aspect ratio around 0.83. The standard deviation is reported as the error bar. Average values are calculated over a population of 23 and 29 cells for inner and outer regions, respectively. The black dashed lines show the average velocities.

Cell motion was further characterized by calculating the radial (*V*_*ρ*_), and angular (*ω*) velocity of the cells moving in the two regions over a time period of 12 h. The velocities in polar coordinates were calculated considering the centre of the cluster as the centre of the polar coordinate. These are plotted as a function of time in figure 6*a*,*b*, respectively, for the cells in the outer region, and in figure 6*c*,*d*, for the cells in the core of the cluster.

Our data suggest that the movement along the radial direction of the cells in the outer as well as in the inner region of the colony is quite limited, which is consistent with a free expansion regime. Concerning the radial velocity, by looking at different clusters an average value of 0.04 μm min^−1^ can be obtained (which was used above to estimate the transition between the free growth and the proliferation inhibition regimes). Hence, the cells migrating along the edge of the aggregate do not invade the core region, and vice versa.

**Figure 7.**
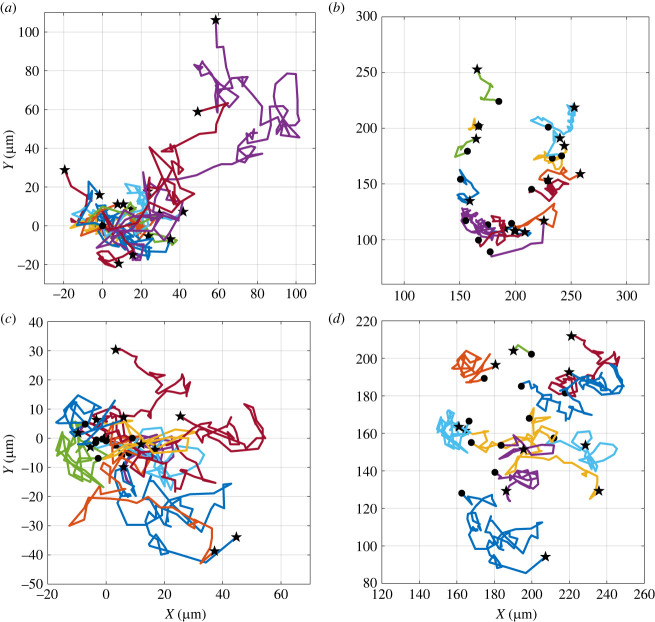
Trajectories of the cells moving in the outer (*a*,*b*) and inner (*c*,*d*) region of the cluster with an average radius of 70 μm and an aspect ratio around 0.65. Solid circles (stars) show the starting (end) points of the trajectory. For the outer (inner) region of cells the average is taken over 17 (11) cells.

The cells moving in the outer region of the cluster show higher tangential velocity compared to the cells in the core, and display a higher coordination in the rotatory movement as suggested by the lower amplitude of the error bars. The time evolution of the angular velocity of the cells on the edge and in the core region of the cluster exhibits the same trend, suggesting that the outer cells drag the inner ones, and coordinate their rotatory movement. Overall, the cells of the entire cluster move in a concerted fashion like a rotating disc. The coordinated rotational movement of the cells in colonies with a round shape has been already shown in previous works [36,59], but only for cells confined in micropatterns. To the best of our knowledge, this is the first report of a systematic study of CAM in freely expanding cell colonies. Since clusters exhibit different shapes, which can have an effect on coordinated cell rotation, we repeated the tracking analysis of cell positions in a colony with an average radius of 70 μm and an aspect ratio 0.65, i.e. with a less circular shape. In this case too, the cluster was divided into two regions, the inner one being a circle with a radius of 38 μm. In figure 7, the trajectories of the cells moving in the outer (*a*,*b*) and inner (*c*,*d*) region are plotted relative to a common origin (figure 7*a*,*c*) or to their actual initial position (figure 7*b*,*d*). The plots in figure 7 show that the cell trajectories are less coherent with respect to the ones of a more circular cluster (see figure 7).

**Figure 8.**
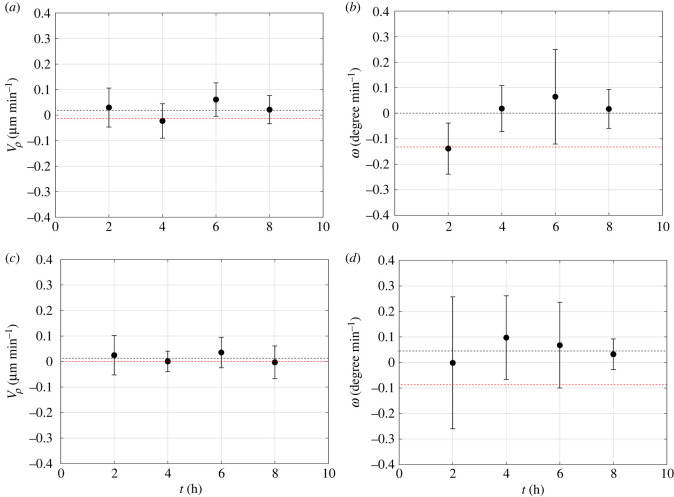
Evolution in time of the radial (*V*_*ρ*_), and angular (*ω*) velocity of the cells moving in the outer (*a*,*b*) and inner (*c*,*d*) region of a cluster with an average radius of 70 μm and an aspect ratio around 0.65. The standard deviation is reported as error bar. For the outer (inner) region of cells the average is taken over 17 (11) cells. The black dashed lines show the average velocities. The red dashed lines have been added from figure 6 for comparison.

Such a result is confirmed by the radial (*V*_*ρ*_), and angular (*ω*) velocity of the cells moving in the outer (figure 8*a*,*b*) and inner (figure 8*c*,*d*) regions of the cluster. The angular velocities of both the outer and the inner region have a time average quite close to 0, thus showing that a coordinated rotation is very small, if any at all. Furthermore, the error bars of the angular velocities (figure 8*c*,*d*) are larger than the ones of the more circular cluster of figures 5 and 6. This result shows a more random motility in the less circular cluster.

**Figure 9.**
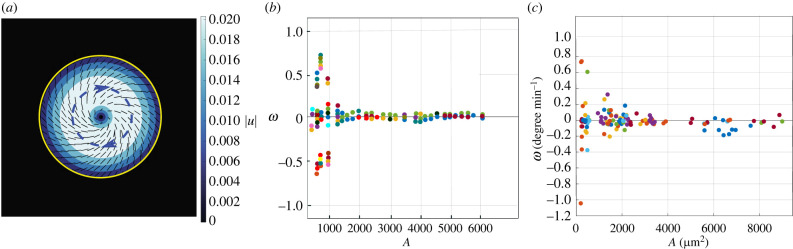
(*a*) A rotating colony in simulations. Colour shows the magnitude of the velocity field, blue dashed arrows show the direction of the rotation and yellow shows the interface of the colony. (*b*,*c*) Angular cell velocity as a function of cluster area in simulations and experiments, respectively. To compare the rotational velocity with the experimental data, we matched the initial area and angular velocity in simulations with the initial area and angular velocity in the experiment and rescaled the axes accordingly. In both cases, colonies show collective rotation for intermediate values of the droplet area.

It is tempting to conjecture that boundary curvature variations in a more irregular (non-circular) cluster lead to non-uniform motile behaviour of the cells. This finding, combined with a lack of radial motion independently of cluster shape (which shows that cells tend to stay within a defined radial region inside each cluster), can be explained by the effect of cell–cell junctions, which hold them together, thus generating a solid-body rotation. Irregular cluster shapes are mostly generated by the merging of different clusters when they get in contact owing to their growth. However, no systematic trend of aspect ratio versus cluster size was found (data not shown for the sake of brevity). The effect of cluster size on rotation is illustrated in figure 9, where the average angular cell velocity in a cluster is plotted as a function of cluster area for 30 cell clusters from two independent experiments.

The continuous line is a linear fit of the data and is coincident with the horizontal axis, thus showing that the average angular cell velocity among all the clusters is zero. However, the absolute value of angular velocity is a decreasing function of cluster size and becomes essentially zero at a value of cluster area of about 3000 μm^2^, which corresponds to a diameter of 60 μm. This dependence of the angular velocity on *A*_cluster_ is in qualitative agreement with the results from the literature on MDCK cells in micropatterned substrata [36], where the collective rotation was found to be persistent at higher cell densities, but with a decrease of the average cell velocity with cluster size. Interestingly, in this previous study the synchronized collective rotation was only observed in 100 and 200 μm diameter regions (close to the correlation length found in unconfined MDCK epithelial sheets [60]), while transient vortices reminiscent of active turbulence were found in larger regions. The lower value of the critical size for coordinated cell rotation observed in our experiments (60 versus 200 μm) can be owing to the different cell line (T84 versus MDCK) and/or to the free versus confined expansion regime. Another interesting feature of the data in figure 9*c* is that clockwise (negative) and counterclockwise (positive) rotations are equally likely, since the average angular velocity is zero. This is not a trivial result, since some asymmetry in collective rotation, with a switch in CAM direction from time to time, has been observed in confluent MDCK cells elsewhere [25] and attributed to cell chirality, which is a phenotype-specific property assuming a clockwise value for several (but not all) cell types from fibroblast to endothelial cells [61]. Our data do not support a special orientation in cell rotation for the T84 cell line under investigation.

## 4. Simulation results

We next checked whether the cell rotation could be described in terms of the theories of active nematics. We used the continuum active nematohydrodynamic equations of motion (see Methods) to consider growing, initially isotropic cell colonies. We found that, once the size of the colony has reached a threshold value, active flows induced nematic order and the colonies could start rotating (see figure 9). The angular velocity in simulations with different colony areas is shown in figure 9*b* and can be compared with the experimental results in figure 9*c*. We found that smaller colonies show collective rotation and the angular velocity decreases with colony area and is zero for larger colonies. The figure also shows that the direction of the rotation is randomly selected between clockwise and anticlockwise, and does not change over time, in agreement with the experiments, and as expected as the active stress does not have any chirality. In the simulations, for very small colonies, we do not observe rotation. This is because for very small colonies the total activity needed for the formation of the nematic order in the colony is not enough, and elasticity stabilizes the isotropic phase.

Agent-based [62] and continuum [63] simulations in polar systems have also observed stable rotating phases in colonies. In [63], a polarity–velocity alignment was assumed. Moreover, it was assumed that cells at the edge zone are radially polarized and motile, whereas cells in the bulk of the tissue are unpolarized and non-motile. In [64], an analytical solution for active viscoelastic materials made of polar filaments was presented. It is known that, in polar systems, the lowest order charge for a topological defect is ±1. As a result, the authors focus on defects with charge equal to one. They show that in equilibrium four types of defects with charge one are possible. These are: two asters and two vortices. When the system is active, formation of spiral defects is also possible. By contrast to asters and vortices, the spiral defects in active systems show rotation. This is similar to our simulations where we found a spiral defect in active droplets. The difference is that our system is nematic, and as a result the lowest energy topological charges in our system are ±1/2 defects. However, the shear flow leads to the formation of +1 spiral defects. Polar forces can be important at the colony interface where the effect of contact inhibition of locomotion is small. Inside the colony, however, the contact inhibition of locomotion becomes important and it is more likely that cells exert nematic forces mediated by junctions with their neighbouring cells. Our continuum model confirms that nematic forces are sufficient for the formation of rotating phases in cell colonies. We should note, however, that since we ignore polar forces in our model, we cannot study any polar patterns that can appear at the interface of the colony.

## 5. Conclusion

In this work, we show for the first time, to our knowledge, that coordinated angular motion is a feature of freely expanding epithelial cells and does not require spatial confinement of the cells on micropatterned substrata. One of the main implications of this result is that physical confinement of cells at the boundary of a cluster is not a necessary condition for the development of CAM. This does not rule out a possible role of boundary cells to initiate a coherent rotation that propagates to the inner parts of a cluster. A further argument supporting a special status of boundary cells is their morphology (more flattened and with several protrusions exploring the surrounding environment), which is quite different with respect to the one exhibited by cells inside a cluster. The presence of boundary cells forming finger-like structures has been associated with symmetry breaking in expanding circular cell colonies by Comelles *et al.* [40]. We show indeed that the observed experimental trends are in excellent agreement with predictions based on active nematic theories. In particular, we found, both by experiments and simulations, that the angular velocity was a decreasing function of cluster size. The spontaneous tendency of epithelial cells to rotate in a synchronized fashion could reflect a morphogenetic mechanism, such as the formation of acini in the mammary glands and the tangential cell orientation found on the wall of intestinal microvilli. More work is needed to unravel these mechanisms and elucidate the drivers of coordinated cell rotation at the molecular scale.

## Data Availability

The code is available on request. The data are provided in electronic supplementary material [65].

## References

[1] Rørth P. 2012 Fellow travellers: emergent properties of collective cell migration. EMBO Rep. **13**, 984-991. (10.1038/embor.2012.149) 23059978 PMC3492716

[2] Vedel S, Tay S, Johnston DM, Bruus H, Quake SR. 2013 Migration of cells in a social context. Proc. Natl Acad. Sci. USA. **110**, 129-134. (10.1073/pnas.1204291110) 23251032 PMC3538227

[3] Friedl P, Noble PB, Walton PA, Laird DW, Chauvin PJ, Tabah RJ, Black M, Zänker KS. 1995 Migration of coordinated cell clusters in mesenchymal and epithelial cancer explants *in* *vitro*. Cancer Res. **55**, 4557-4560. 7553628

[4] Friedl P, Gilmour D. 2009 Collective cell migration in morphogenesis, regeneration and cancer. Nat. Rev. Mol. **10**, 445-457. (10.1038/nrm2720) 19546857

[5] Méhes E, Vicsek T. 2014 Collective motion of cells: from experiments to models. Integr. Biol. **6**, 831-854. (10.1039/C4IB00115J) 25056221

[6] Rørth P. 2009 Collective cell migration. Annu. Rev. Cell Dev. Biol. **25**, 407-429. (10.1146/annurev.cellbio.042308.113231) 19575657

[7] Friedl P, Locker J, Sahai E, Segall JE. 2012 Classifying collective cancer cell invasion. Nat. Cell Biol. **14**, 777-783. (10.1038/ncb2548) 22854810

[8] Ilina O, Friedl P. 2009 Mechanisms of collective cell migration at a glance. J. Cell Sci. **122**, 3203-3208. (10.1242/jcs.036525) 19726629

[9] Rørth P. 2007 Collective guidance of collective cell migration. Trends Cell Biol. **17**, 575-579. (10.1016/j.tcb.2007.09.007) 17996447

[10] Ravasio A et al. 2015 Regulation of epithelial cell organization by tuning cell–substrate adhesion. Integr. Biol. **7**, 1228-1241. (10.1039/C5IB00196J) PMC5423524 26402903

[11] Trepat X, Wasserman MR, Angelini TE, Millet E, Weitz DA, Butler JP, Fredberg JJ. 2009 Physical forces during collective cell migration. Nat. Phys. **5**, 426-430. (10.1038/nphys1269)

[12] Ladoux B. 2009 Cells guided on their journey. Nat. Phys. **5**, 377-378. (10.1038/nphys1281)

[13] Ladoux B, Nicolas A. 2012 Physically based principles of cell adhesion mechanosensitivity in tissues. Rep. Prog. Phys. **75**, 116601. (10.1088/0034-4885/75/11/116601) 23085962

[14] Beaune G et al. 2014 How cells flow in the spreading of cellular aggregates. Proc. Natl Acad. Sci. USA **111**, 8055-8060. (10.1073/pnas.1323788111) 24835175 PMC4050549

[15] Gompper G et al. 2020 The 2020 motile active matter roadmap. J. Phys. Condens. Matter **32**, 193001. (10.1088/1361-648X/ab6348) 32058979

[16] Mueller R, Yeomans JM, Doostmohammadi A. 2019 Emergence of active nematic behavior in monolayers of isotropic cells. Phys. Rev. Lett. **122**, 048004. (10.1103/PhysRevLett.122.048004) 30768306

[17] Angelini TE, Hannezo E, Trepat X, Fredberg JJ, Weitz DA. 2010 Cell migration driven by cooperative substrate deformation patterns. Phys. Rev. Lett. **104**, 168104. (10.1103/PhysRevLett.104.168104) 20482085 PMC3947506

[18] Zorn ML, Marel AK, Segerer FJ, Rädler JO. 2015 Phenomenological approaches to collective behavior in epithelial cell migration. Biochim. Biophys. Acta Mol. Cell Res. **1853**, 3143-3152. (10.1016/j.bbamcr.2015.05.021) 26028592

[19] Tanner K, Mori H, Mroue R, Bruni-Cardoso A, Bissell MJ. 2012 Coherent angular motion in the establishment of multicellular architecture of glandular tissues. Proc. Natl Acad. Sci. USA **109**, 1973-1978. (10.1073/pnas.1119578109) 22308439 PMC3277511

[20] Ferrari A, Veligodskiy A, Berge U, Lucas MS, Kroschewski R. 2008 ROCK-mediated contractility, tight junctions and channels contribute to the conversion of a preapical patch into apical surface during isochoric lumen initiation. J. Cell Sci. **121**, 3649-3663. (10.1242/jcs.018648) 18946028

[21] Marmaras A, Berge U, Ferrari A, Kurtcuoglu V, Poulikakos D, Kroschewski R. 2010 A mathematical method for the 3D analysis of rotating deformable systems applied on lumen-forming MDCK cell aggregates. Cytoskeleton **67**, 224-240. (10.1002/cm.20438) 20183868

[22] Vasiev B, Balter A, Chaplain M, Glazier JA, Weijer CJ. 2010 Modeling gastrulation in the chick embryo: formation of the primitive streak. PLoS ONE **5**, e10571. (10.1371/journal.pone.0010571) 20485500 PMC2868022

[23] Huang S, Brangwynne C, Parker K, Ingber DE. 2005 Symmetry-breaking in mammalian cell cohort migration during tissue pattern formation: role of random-walk persistence. Cell. Motil. Cytoskelet. **61**, 201-213. (10.1002/cm.20077) 15986404

[24] Vedula SR, Leong MC, Lai TL, Hersen P, Kabla AJ, Lim CT, Ladoux B. 2012 Emerging modes of collective cell migration induced by geometrical constraints. Proc. Natl Acad. Sci. USA **109**, 12 974-12 979. (10.1073/pnas.1119313109) PMC3420172 22814373

[25] Deforet M, Hakim V, Yevick H, Duclos G, Silberzan P. 2014 Emergence of collective modes and tri-dimensional structures from epithelial confinement. Nat. Commun. **5**, 1-9. (10.1038/ncomms4747) 24796352

[26] Marel AK, Zorn M, Klingner C, Wedlich-Söldner R, Frey E, Rädler JO. 2014 Flow and diffusion in channel-guided cell migration. Biophys. J. **107**, 1054-1064. (10.1016/j.bpj.2014.07.017) 25185541 PMC4156682

[27] Peyret G, Mueller R, Begnaud S, Marcq P, Mège RM, Yeomans JM, Doostmohammadi A, Ladoux B. 2019 Sustained oscillations of epithelial cell sheets. Biophys. J. **117**, 464-478. (10.1016/j.bpj.2019.06.013) 31307676 PMC6697349

[28] Caserta S, Campello S, Tomaiuolo G, Sabetta L, Guido S. 2013 A methodology to study chemotaxis in 3-D collagen gels. AlChE J. **59**, 4025-4035. (10.1002/aic.14164)

[29] Ascione F, Caserta S, Guido S. 2017 The wound healing assay revisited: a transport phenomena approach. Chem. Eng. Sci. **160**, 200-209. (10.1016/j.ces.2016.11.014)

[30] Kim JH et al. 2013 Propulsion and navigation within the advancing monolayer sheet. Nat. Mater. **12**, 856-863. (10.1038/nmat3689) 23793160 PMC3750079

[31] Trepat X, Fredberg JJ. 2011 Plithotaxis and emergent dynamics in collective cellular migration. Trends Cell Biol. **21**, 638-646. (10.1016/j.tcb.2011.06.006) 21784638 PMC3202659

[32] Xu H, Nejad MR, Yeomans JM, Wu Y. 2022 Geometrical control of interface patterning underlies active matter invasion. *arXiv* (http://arxiv.org/abs/2208.12424).

[33] Doostmohammadi A, Thampi SP, Saw TB, Lim CT, Ladoux B, Yeomans JM. 2015 Celebrating Soft Matter’s 10th Anniversary: cell division: a source of active stress in cellular monolayers. Soft Matter **11**, 7328-7336. (10.1039/C5SM01382H) 26265162

[34] Prost J, Jülicher F, Joanny JF. 2015 Active gel physics. Nat. Phys. **11**, 111-117. (10.1038/nphys3224)

[35] Doostmohammadi A, Thampi SP, Yeomans JM. 2016 Defect-mediated morphologies in growing cell colonies. Phys. Rev. Lett. **117**, 048102. (10.1103/PhysRevLett.117.048102) 27494503

[36] Doxzen K, Vedula SR, Leong MC, Hirata H, Gov NS, Kabla AJ, Ladoux B, Lim CT. 2013 Guidance of collective cell migration by substrate geometry. Integr. Biol. **5**, 1026-1035. (10.1039/c3ib40054a) 23784144

[37] Doostmohammadi A, Yeomans JM. 2019 Coherent motion of dense active matter. Eur. Phys. J.: Spec. Top. **227**, 2401-2411. (10.1140/epjst/e2019-700109-x)

[38] Segerer FJ, Thüroff F, Alberola AP, Frey E, Rädler JO. 2015 Emergence and persistence of collective cell migration on small circular micropatterns. Phys. Rev. Lett. **114**, 228102. (10.1103/PhysRevLett.114.228102) 26196648

[39] Siedlik MJ, Manivannan S, Kevrekidis IG, Nelson CM. 2017 Cell division induces and switches coherent angular motion within bounded cellular collectives. Biophys. J. **112**, 2419-2427. (10.1016/j.bpj.2017.05.001) 28591614 PMC5474845

[40] Comelles J, Soumya SS, Lu L, Le Maout E, Anvitha S, Salbreux G, Jülicher F, Inamdar MM, Riveline D. 2021 Epithelial colonies *in vitro* elongate through collective effects. eLife **10**, e57730. (10.7554/eLife.57730) 33393459 PMC7850623

[41] Puliafito A, Hufnagel L, Neveu P, Streichan S, Sigal A, Fygenson DK, Shraiman BI. 2012 Collective and single cell behavior in epithelial contact inhibition. Proc. Natl Acad. Sci. USA **109**, 739-744. (10.1073/pnas.1007809109) 22228306 PMC3271933

[42] Silano M et al. 2012 Early tissue transglutaminase–mediated response underlies K562 (S)-cell gliadin-dependent agglutination. Pediatr. Res. **71**, 532-538. (10.1038/pr.2012.4) 22314661

[43] Ascione F, Caserta S, Perris R, Guido S. 2014 Investigation of cell dynamics in vitro by time lapse microscopy and image analysis. Chem. Eng. Trans. **38**, 517-522.

[44] Vasaturo A, Caserta S, Russo I, Preziosi V, Ciacci C, Guido S. 2012 A novel chemotaxis assay in 3-D collagen gels by time-lapse microscopy. PLoS ONE **7**, e52251. (10.1371/journal.pone.0052251) 23284956 PMC3526591

[45] Saw TB et al. 2017 Topological defects in epithelia govern cell death and extrusion. Nature **544**, 212-216. (10.1038/nature21718) 28406198 PMC5439518

[46] Saw TB, Xi W, Ladoux B, Lim CT. 2018 Biological tissues as active nematic liquid crystals. J. Adv. Mater. **30**, 1802579. (10.1002/adma.201802579) 30156334

[47] Guillamat P, Blanch-Mercader C, Pernollet G, Kruse K, Roux A. 2022 Integer topological defects organize stresses driving tissue morphogenesis. Nat. Mater. **21**, 588-597. (10.1038/s41563-022-01194-5) 35145258 PMC7612693

[48] Nejad MR, Yeomans JM. 2022 Active extensile stress promotes 3D director orientations and flows. Phys. Rev. Lett. **128**, 048001. (10.1103/PhysRevLett.128.048001) 35148135

[49] Thijssen K, Nejad MR, Yeomans JM. 2020 Role of friction in multidefect ordering. Phys. Rev. Lett. **125**, 218004. (10.1103/PhysRevLett.125.218004) 33275020

[50] Santhosh S, Nejad MR, Doostmohammadi A, Yeomans JM, Thampi SP. 2020 Activity induced nematic order in isotropic liquid crystals. J. Stat. Phys. **180**, 699-709. (10.1007/s10955-020-02497-0)

[51] Marenduzzo D, Orlandini E, Cates ME, Yeomans JM. 2007 Steady-state hydrodynamic instabilities of active liquid crystals: hybrid lattice Boltzmann simulations. Phys. Rev. E **76**, 031921. (10.1103/PhysRevE.76.031921) 17930285

[52] Mackay F, Toner J, Morozov A, Marenduzzo D. 2020 Darcy’s law without friction in active nematic rheology. Phys. Rev. Lett. **124**, 187801. (10.1103/PhysRevLett.124.187801) 32441954

[53] Nejad MR, Doostmohammadi A, Yeomans JM. 2021 Memory effects, arches and polar defect ordering at the cross-over from wet to dry active nematics. Soft Matter **17**, 2500-2511. (10.1039/D0SM01794A) 33503081

[54] Méhes E, Mones E, Németh V, Vicsek T. 2012 Collective motion of cells mediates segregation and pattern formation in co-cultures. PLoS ONE **7**, e31711. (10.1371/journal.pone.0031711) 22359617 PMC3280994

[55] Ouaknin GY, Bar-Yoseph PZ. 2009 Stochastic collective movement of cells and fingering morphology: no maverick cells. Biophys. J. **97**, 1811-1821. (10.1016/j.bpj.2009.05.064) 19804711 PMC2756401

[56] Douezan S, Brochard-Wyart F. 2012 Active diffusion-limited aggregation of cells. Soft Matter **8**, 784-788. (10.1039/C1SM06399E)

[57] Qin L, Yang D, Yi W, Cao H, Xiao G. 2021 Roles of leader and follower cells in collective cell migration. Mol. Biol. Cell **32**, 1267-1272. (10.1091/mbc.E20-10-0681) 34184941 PMC8351552

[58] Blow ML, Thampi SP, Yeomans JM. 2014 Biphasic, lyotropic, active nematics. Phys. Rev. Lett. **113**, 248303. (10.1103/PhysRevLett.113.248303) 25541809

[59] Malet-Engra G, Yu W, Oldani A, Rey-Barroso J, Gov NS, Scita G, Dupré L. 2015 Collective cell motility promotes chemotactic prowess and resistance to chemorepulsion. Curr. Biol. **25**, 242-250. (10.1016/j.cub.2014.11.030) 25578904

[60] Poujade M, Grasland-Mongrain E, Hertzog A, Jouanneau J, Chavrier P, Ladoux B, Buguin A, Silberzan P. 2007 Collective migration of an epithelial monolayer in response to a model wound. Proc. Natl Acad. Sci. USA **104**, 15 988-15 993. (10.1073/pnas.0705062104) 17905871 PMC2042149

[61] Wan LQ, Ronaldson K, Park M, Taylor G, Zhang Y, Gimble JM, Vunjak-Novakovic G. 2011 Micropatterned mammalian cells exhibit phenotype-specific left-right asymmetry. Proc. Natl Acad. Sci. USA **108**, 12 295-12 300. (10.1073/pnas.1103834108) 21709270 PMC3145729

[62] Copenhagen K, Malet-Engra G, Yu W, Scita G, Gov N, Gopinathan A. 2018 Frustration-induced phases in migrating cell clusters. Sci. Adv. **4**, eaar8483. (10.1126/sciadv.aar8483) 30214934 PMC6135545

[63] Heinrich MA, Alert R, LaChance JM, Zajdel TJ, Košmrlj A, Cohen DJ. 2020 Size-dependent patterns of cell proliferation and migration in freely-expanding epithelia. eLife **9**, e58945. (10.7554/eLife.58945) 32812871 PMC7498264

[64] Kruse K, Joanny JF, Jülicher F, Prost J, Sekimoto K. 2004 Asters, vortices, and rotating spirals in active gels of polar filaments. Phys. Rev. Lett. **92**, 078101. (10.1103/PhysRevLett.92.078101) 14995891

[65] Ascione F, Caserta S, Esposito S, Villella VR, Maiuri L, Nejad MR, Doostmohammadi A, Yeomans JM, Guido S. 2023 Collective rotational motion of freely-expanding T84 epithelial cell colonies. *Figshare*. (10.6084/m9.figshare.c.6412329) PMC9943890 36872917

