# Correction to: ‘Collective rotational motion of freely expanding T84 epithelial cell colonies’ (2023) by Ascione *et al.*

**DOI:** 10.1098/rsif.2023.0114

**Published:** 2023-03-22

**Authors:** Flora Ascione, Sergio Caserta, Speranza Esposito, Valeria Rachela Villella, Luigi Maiuri, Mehrana R. Nejad, Amin Doostmohammadi, Julia M. Yeomans, Stefano Guido

*J. R. Soc. Interface* **20**, 20220719. (Published online 22 February 2023) (https://doi.org/10.1098/rsif.2022.0719)

The correct list of authors for ‘Collective rotational motion of freely-expanding T84 epithelial cell colonies’ – https://doi.org/10.1098/rsif.2022.0719 – should read Flora Ascione, Sergio Caserta, Speranza Esposito, Valeria Rachela Villella, Luigi Maiuri, Mehrana R. Nejad, Amin Doostmohammadi, Julia M. Yeomans, Stefano Guido.

